# Safe Perinatal Management of Neonates Born to SARS-CoV-2 Positive Mothers at the Epicenter of the Italian Epidemic

**DOI:** 10.3389/fped.2020.565522

**Published:** 2020-10-29

**Authors:** Giacomo Biasucci, Giuseppe Cannalire, Akamin Raymond, Maria Elena Capra, Belinda Benenati, Giovanni Vadacca, Roberta Schiavo, Cristiana Pavesi, Renza Bonini

**Affiliations:** ^1^Pediatrics & Neonatology Unit, Guglielmo da Saliceto Hospital, Piacenza, Italy; ^2^Department of ClinicalPathology, Guglielmo da Saliceto Hospital, Piacenza, Italy; ^3^Obstetrics & Gynecology Unit, Guglielmo da Saliceto Hospital, Piacenza, Italy

**Keywords:** vertical infection transmission, pregnancy, perinatal management, COVID-19 epidemic, breastfeeding (BF), SARS—CoV-2

## Abstract

**Introduction:** 2019-novel Coronavirus Disease (COVID-19) pandemic has recently struck Northern Italy. Limited data are available about COVID-19 during pregnancy and infancy, mostly from China. Herein, our experience on a safe perinatal management of neonates born to COVID-19 mothers is reported.

**Method:** Since late February through May 15, 2020, 375 pregnant women delivered at our City Hospital in Piacenza, at the epicenter of the Italian epidemic. Of these, 144 were tested via a SARS-CoV-2 quantitative rRT-PCR nasopharyngeal swab prior to delivery, firstly on the basis of epidemiological and clinical criteria, then adopting a universal screening approach. All newborns from SARS-CoV-2 positive mothers were tested via nasopharyngeal swab at birth, on day 3 and/or day 7. In case of positive result, they were re-tested on day 14.

**Results:** Fifteen women tested positive for SARS-CoV-2 infection. All newborns except one were born at term. All of them were non-infected at birth, irrespective of mode of delivery; 13 out 15 remained negative; the two positive neonates became negative by day 14 of life. All of them have always remained asymptomatic. All newborns except two were allowed to have immediate bonding, permanent rooming-in, and direct breastfeeding.

**Conclusions:** Our study supports the claim that COVID-19 in pregnancy is not associated with worse clinical outcomes compared to non-COVID-19 pregnant women and/or with higher rates of preterm birth and intrauterine growth restriction. Intrauterine vertical transmission of SARS-CoV-2 seems to be unlikely. Breastfeeding appears to be safe and protective for the neonate, once appropriate preventive measures are adopted.

## Introduction

The recent pandemic caused by a novel Coronavirus (SARS-CoV-2) first isolated in Wuhan (Hubei Region, China) in December 2019 is still inflicting heavy consequences on public health in most parts of the world, as 2019-novel Coronavirus Disease (COVID-19) has rapidly spread worldwide. Major clinical features range from the common cold and general malaise (flu-like syndrome) to high fever, cough, severe interstitial pneumonia, microvascular lung vessels obstructive thrombo-inflammatory syndrome, respiratory failure, and death. Other manifestations include myalgia, headache, anosmia, ageusia and diarrhea. Specific abnormalities on chest radiographic imaging (e.g., ground glass opacities), leukopenia, lymphopenia, and thrombocytopenia have also been reported (1–3). No specific effective treatment or vaccine is yet available (3–5).

Despite an increasing number of scientific papers focusing on pathophysiological aspects of the disease and the potential efficacy of different antiviral and/or anti-inflammatory drugs, limited data are available in literature about COVID-19 during pregnancy and infancy, most of them from studies in China. Indeed, among other clinical and public health issues, the management of a COVID-19 mother and her offspring is of major concern. According to some studies focusing on previous epidemics (6–9), pregnant women seem to be at high risk of developing viral infections, such as influenza-A, H1N1 virus, Severe Acute Respiratory Syndrome Coronavirus (SARS-CoV) and Middle East Respiratory Syndrome Coronavirus (MERS-CoV), and appear to have worse clinical outcomes in terms of maternal mortality, spontaneous abortion, and preterm delivery compared to non-infected pregnant women. Indeed, as for the current epidemic, in a very recent study COVID-19 severity in pregnant women has been reported as 86% mild, 9.3% severe and 4.7% critical, thus appearing to be similar to that of non-pregnant adults (10). Yan et al. (11) in another recent retrospective study from China, describing the clinical characteristics and pregnancy outcomes in 116 pregnant women, confirmed that SARS-CoV-2 infection has the same clinical features in pregnant women as in the general female non-pregnant population. The authors also demonstrated that SARS-CoV-2 infection during pregnancy is not associated with an increased risk of spontaneous abortion and spontaneous preterm birth. However, more data about the clinical features of COVID-19 during pregnancy are needed to draw any definitive conclusion.

Which mode of delivery can be considered safer in the perinatal management of COVID-19 pregnant women represents another major question. An Italian retrospective study described the intrapartum management of 42 COVID-19 women. Three out of 42 newborns tested positive for SARS-CoV-2: two newborns probably acquired the infection in the postpartum period, and one newborn was contaminated during an operative delivery. The results of this study suggest that vaginal delivery is associated with a low risk of intrapartum transmission of SARS-CoV-2 infection to the newborn (12).

Since February 22, 2020, we have started to deal with the perinatal management of COVID-19 pregnant women, shortly after the epidemic outbreak in Wuhan, China, and right after the first Italian COVID-19 patient was identified in Codogno, a small town located at the southern part of Lombardy region. Since then, SARS-CoV-2 infection has rapidly spread in the neighboring areas, strongly affecting northern Italian regions, mostly Lombardy and Emilia-Romagna. As the city of Piacenza is located at the northwestern end of Emilia-Romagna region, right on the border with Lombardy, it has been immediately hit by the Italian outbreak, becoming a major epicenter of the epidemic and one the most affected Italian cities with respect to the number of infected people and deaths per total population.

Herein, our experience on perinatal management of 15 neonates born to COVID-19 mothers in Piacenza is described and compared with data reported in literature to date.

## Method

Since February 22, (outbreak onset) to April 23, 2020, we prospectively tested all pregnant women referring to the Labor Unit at Guglielmo da Saliceto City Hospital in Piacenza via a SARS-CoV-2 quantitative reverse transcriptase real-time polymerase chain reaction (rRT-PCR) nasopharyngeal swab, on the basis of the following epidemiological and clinical criteria: having had close contact with SARS-CoV-2 infected subjects, or traveled to COVID-19 exposed countries during the previous 14 days; being a healthcare professional working in a COVID-19 dedicated area; presenting with fever (> 37.5°C), cough and/or dyspnea. Thereafter, we universally screened for SARS-Cov-2 infection all pregnant women referring to our Labor Unit. All COVID-19 mothers were identified and managed in a dedicated and isolated area. In our City Hospital, we assist preterm deliveries from 30 weeks of gestational age (GA) onwards, as we do not have a Neonatal Intensive Care Unit. We serve a population of almost 300.000 people, with about 2,000 deliveries per year. Though severely sick COVID-19 pregnant women have been planned to be transferred to a tertiary level Hospital, no SARS-CoV-2 positive pregnant woman required to be transferred to other Hospitals during the study period.

Asymptomatic and paucisymptomatic COVID-19 mothers were not separated from their offspring, both sharing a dedicated and isolated room. At the same time, breastfeeding and/or expressed breast milk feeding was encouraged and strict compliance with appropriate hygiene standards was required. Thus, mothers were also asked to wear surgical mask while breastfeeding, and infants were kept at a safe distance (>2 m) for the rest of the time. Skilled midwives constantly monitored maternal adherence to these recommendations. Mother and newborn were separated only in case of maternal severe clinical symptoms.

All newborns from SARS-CoV-2 positive mothers were tested via a SARS-CoV-2 quantitative rRT-PCR nasopharyngeal swab at birth, on day 3 and/or day 7 during their hospital stay. In case of positive result, neonates were re-tested on day 14.

In accordance with family pediatricians, we agreed to monitor babies of COVID-19 mothers throughout the first postnatal week, unless specifically agreed otherwise. At least 2 weeks after babies' discharge from the hospital, parents were asked to report us on their clinical outcome by a phone interview.

Infants' information including gender, GA at birth, birth weight, postnatal adaptation, clinical symptoms, eventually laboratory and radiological parameters was also prospectively collected.

## Results

Since the Italian outbreak onset in late February through May 15, 375 pregnant women have given birth to 377 babies at Guglielmo da Saliceto City Hospital in Piacenza. From February 22 to April 23 we tested 32 pregnant women for SARS-CoV-2 out of 263 referring to our Labor Unit; 10 of them tested positive. Since the start of universal screening on April 24, another 112 pregnant women have been tested and 5 were found to be positive for SARS-CoV-2 infection.

Thus, we overall identified and managed 15 SARS-CoV-2 positive mothers and their newborns out of 144 tested women. Four pregnant women presented with fever: of these, two had also cough and dyspnea and two just cough; the remaining 11 were asymptomatic. Cesarean section (CS) accounted for six out of 15 deliveries: five were due to obstetrical reasons (previous CS, fetal breech presentation and decels of fetal heartbeat), and one to maternal clinical condition related to SARS-CoV-2 infection (see Table 1).

**Table 1:** Maternal characteristics.

**Case #**	**Gestational age (week)**	Mode of delivery^*^	**Rooming-in and Breastfeeding**	**Age (years)**	**Symptoms on admission**	**Swab after 2 weeks**
1	40^+2^	CS	No	41	Fever, Cough, Dyspnea	Neg
2	39	CS	Yes	32	Fever, Cough	Neg
3	39^+4^	Vaginal	No	24	Fever, Cough, Dyspnea	Neg
4	41	Vaginal	Yes	29	Asymptomatic	Neg
5	36^+4^	CS	Yes	30	Asymptomatic	Neg
6	40^+1^	Vaginal	Yes	31	Asymptomatic	Neg
7	40^+6^	Vaginal	Yes	37	Asymptomatic	Neg
8	40^+1^	Vaginal	Yes	28	Asymptomatic	Neg
9	39	CS	Yes	28	Fever, Cough	Neg
10	39^+1^	Vaginal	Yes	25	Asymptomatic	Neg
11	38^+2^	CS	Yes	28	Asymptomatic	Neg
12	40^+6^	Vaginal	Yes	29	Asymptomatic	Neg
13	40^+3^	Vaginal	Yes	32	Asymptomatic	Neg
14	38^+3^	CS	Yes	38	Asymptomatic	Neg
15	39^+6^	Vaginal	Yes	31	Asymptomatic	Neg

* *Causes for CS delivery: # 1: Covid-19 maternal clinical symptoms; # 2: fetal breech presentation; # 5: fetal decels of heartbeat; # 9,11,14: previous CS*.

All babies except one were born at term; mean GA and birth weight were 39^+6^ ± 8.4 SD (range: 36^+4^- 41) weeks and 3,245 ± 482.38 SD (range: 2,270–4,260) grams, respectively. All of them were adequate for gestation age; gender was distributed as such: nine males, six females. No neonatal respiratory distress was reported; 1st min Apgar score ranged from 8 to 10, whereas it was 10 for all newborns at 5'. Just two out of the 15 neonates tested positive for COVID-19, one on day 3 and one on day 7, both being asymptomatic and directly breastfed. Nevertheless, both neonates tested negative on day 14 (see Table 2). Only our first neonate born to a severely symptomatic COVID-19 mother required immediate isolation. He was then partially formula fed throughout the first 2 weeks of life. As soon as his mother's condition improved, the baby was partially fed with her expressed breast milk. Direct breastfeeding was allowed 2 weeks later, when the mother tested negative for SARS-CoV-2. Except for another baby whose mother was given antiviral drugs not compatible with breastfeeding, all the remaining 13 infants were allowed to have immediate bonding, permanent rooming-in, and direct breastfeeding.

**Table 2:** Neonatal characteristics.

**Case #**	**Sex**	**Birth weight** ** (gr)**	**5'Apgar score**	**Swab** ** at birth**	**Swab** ** (day3)**	**Swab** ** (day7)**	**Swab** ** (day 14)**	**Rooming-in and** ** breast feeding**	**Symptoms in** ** hospital**	**Symptoms after** ** discharge**
1	M	3,320	10	Neg	Neg		Neg	No	None	None
2	M	3,180	10	Neg		Pos	Neg	Yes	None	None
3	F	3,080	10	Neg		Neg		No	None	None
4	F	3,180	10	Neg	Pos	Neg	Neg	Yes	None	None
5	M	2,270	10	Neg		Neg		Yes	None	None
6	F	3,365	10	Neg	Neg	Neg		Yes	None	None
7	M	4,260	10	Neg	Neg	Neg		Yes	None	None
8	M	3,500	10	Neg	Neg	Neg		Yes	None	None
9	F	3,150	10	Neg	Neg	Neg		Yes	None	None
10	M	3,320	10	Neg	Neg	Neg		Yes	None	None
11	F	2,970	10	Neg	Neg	Neg		Yes	None	None
12	M	3,720	10	Neg	Neg	Neg		Yes	None	None
13	M	3,700	10	Neg	Neg	Neg		Yes	None	None
14	F	2,450	10	Neg	Neg	Neg		Yes	None	None
15	M	3,210	10	Neg	Neg	Neg		Yes	None	None

During their hospital stay, all infants remained asymptomatic, with normal temperature and vital parameters. Currently, all the babies have remained asymptomatic and 14/15 are still breastfed. At the same time, all their mothers had already recovered and retested negative for SARS-CoV-2 infection.

## Discussion

During the world pandemic, the first baby of a COVID-19 mother in Europe and, to the best of our knowledge, outside mainland China, was delivered in our City Hospital on February 24th. At that time, the precautionary indications published by Liu et al. (13) and the Center for Disease Control and Prevention (CDC) (14) were in favor of CS delivery and immediate separation of the mother-newborn dyad in symptomatic mothers (15). In the light of these indications, our first COVID-19 mother underwent a CS delivery and her baby was immediately isolated. Shortly after, the Task Force on Breastfeeding of the Italian Ministry of Health, together with experts of the Italian Society of Neonatology, issued the first edition of “ad interim indications,” subsequently updated and published online (16). This document emphasized that if a COVID-19 suspected or affected mother is asymptomatic or paucisymptomatic prior to delivery, rooming-in is feasible and direct breastfeeding is advisable, under strict measures of infection control. If direct breastfeeding is not possible due to the mother's condition, it is advisable to proceed feeding the baby with the mother's expressed breast milk.

Based on data available in the literature, it seems that infants and children may be less vulnerable to SARS-CoV-2 infection and, when affected, symptoms are usually milder than in adult patients (2, 17). Indeed, it has been hypothesized that they may be protected by some antibodies against other Coronaviruses, as well as they may not develop a strong inflammatory reaction which is partially responsible for the lung injury during COVID-19 (18–20). As newborns do not have antibodies against other Coronaviruses, they should theoretically be more vulnerable to SARS-CoV-2 infection. Nevertheless, SARS-CoV-2 has never been detected in amniotic fluid, placenta and cord blood, nor in the breast milk of COVID-19 positive mothers, according to literature to date (8, 11). Moreover, very recent data, showing that angiotensin-converting enzyme-2 receptors have very low expression in the placenta, seem to further confirm that SARS-CoV-2 intrauterine vertical transmission should be unlikely (21). Indeed, whether or not newborns are somehow protected from the risk of vertical transmission is still a matter of debate. With regard to this issue, Dong et al. (22) described an asymptomatic infant girl, delivered by CS and immediately separated from her COVID-19 mother. Soon after birth, the baby's serology tested positive for both SARS-CoV-2 immunoglobulin (Ig) -G and Ig-M titers, despite simultaneous and repeated negative nasopharyngeal swabs. Based on postnatal positive Ig-M titer and negative swab, the authors hypothesized an intrauterine infection. Anyway, due to the absence of SARS-CoV-2 testing of amniotic fluid or placenta, a possible vertical transmission could not be ascertained. Alzamora et al. (23) also speculated on a potential vertical transmission in a very recent paper. They reported on a neonate born prematurely (33 weeks GA) to a severely ill COVID-19 mother, by CS delivery. Due to the infant's postnatal depression, the baby was immediately separated from his mother and breastfeeding was not initiated. Neonatal serology at birth yielded negative Ig-G and Ig-M titers. Nevertheless, his nasopharyngeal swabs, obtained 16 h after delivery and 48 h later, tested positive for SARS-CoV-2. Maternal serology, first reported negative soon after delivery, tested positive for both Ig-G and Ig-M 4 days later. Infant's outcome was consistent with his prematurity and favorable, with constantly negative serology. Although authors raised some suspicion of SARS-CoV-2 intrauterine vertical transmission, they recognized that the lag time to the first nasopharyngeal swab does not permit to rule out a potential perinatal transmission. About the same issue, Zhu et al. (20) have recently described ten neonates, born to suspected COVID-19 mothers, by CS delivery. No information on the type of feeding was provided. All infants developed respiratory symptoms in the first week of life, but all their nasopharyngeal swabs tested negative for SARS-CoV-2, thus not supporting the hypothesis of a vertical transmission. In their report on 116 pregnancies, Yan et al. (11) came to the same conclusion that there was no evidence of a vertical transmission of SARS-CoV-2 infection, especially during the last trimester of pregnancy. In agreement with Kimberlin and Stagno (24) and Simões e Silva and Leal (25) we believe that more definitive evidence is needed to assess whether or not SARS-CoV-2 infection can be acquired *in utero*.

Data from our study support the lack of intrauterine vertical transmission, as none of our newborns tested positive for SARS-CoV-2 at birth, regardless of maternal symptoms and mode of delivery.

As for postnatal clinical follow up and management, Zeng (26) and Wang (27) et al. reported two cases of SARS-CoV-2 infected newborns, diagnosed at 17 and 19 days of age. Both had been infected postnatally, through horizontal transmission, suggesting that more attention should be paid to parents' and caregivers' education about the use of appropriate preventive equipment and safe newborn's management. Breindhal et al. (28) also pointed out that adopting a precautionary approach such as a strict adherence to preventive measures by parents, and the use of personal protective equipment by doctors and nurses is crucial for the newborn's safety.

The potential risk of horizontal transmission of the infection is also evident in our study, albeit only two out of 15 babies tested positive during their hospital stay, both being directly breastfed in a rooming-in setting. As already mentioned, both of them had tested negative at birth and turned to be negative within 1 week. With regard to this aspect, the duration of viral shedding has been reported to be very variable, depending on the severity of the illness, and one study demonstrated that asymptomatic infection was associated with a higher likelihood of nasopharyngeal viral RNA clearance within the first week of diagnosis compared with symptomatic infection (29). Thus, although false positive results on day 3 and 7 in our two asymptomatic newborns cannot be excluded, the subsequent nasopharyngeal negative tests within 1 week may potentially reflect a rapid viral clearance.

## Conclusions

Besides the relatively few COVID-19 neonates described during the Chinese epidemic (30), to the best of our knowledge our experience is among the very few outside China reported in the literature so far. Although based on a limited number of mother-newborn couples, our data support the current World Health Organization (WHO) (31) recommendations encouraging immediate bonding, rooming-in and breastfeeding, at least in case of not severely ill mothers.

We acknowledge that bonding and breastfeeding may expose the newborn to a higher risk of contracting SARS-CoV-2 from the infected mother through contact or droplets, as occurred in two of our asymptomatic babies. However, as reported in the vast majority of early infected infants described in literature so far, including ours, they are not likely to develop serious symptoms. A recent paper by Zeng et al. reported that among 33 neonates of COVID-19 mothers, three presented with respiratory symptoms in the first days of life and tested positive for COVID-19 on day 2 after birth. The only seriously ill neonate in this case series was a 31 week GA preterm infant (32).

Thus, considering that all our newborns, including those who tested positive for COVID-19, remained asymptomatic and in good health, we believe that the benefits of bonding and breastfeeding outweigh the potential risk of horizontal transmission of SARS-CoV-2 infection. Moreover, as during such a pandemic even quarantine itself may represent a very emotionally stressful experience for COVID-19 mothers, we believe that separating mother and neonate as well as precluding direct breastfeeding would even worsen their psychological trauma.

Finally, with regard to the prevention of the potential risk of horizontal transmission of viral infection, our experience supports the evidence to adopt strict hygiene measures such as avoiding kissing the neonate, placing him in a cradle at a safe distance (at least 2 m) from the infected mother, wearing a surgical mask during feeding and intimate contact with the baby, and washing hands before feeding.

We conclude with seven key lessons (Table 3) we learnt from practice on perinatal management of COVID-19 pregnant women and newborns couples, intended to be practical suggestions for those professionals who may have the need to deal with this experience. Even though based on and in agreement with the most recent data, we recognize that our conclusions should be considered “ad interim,” as they might be subject to change in the future, due to the continuous growth of knowledge about the COVID-19 perinatal transmission and clinical features of neonatal SARS-CoV-2 infection.

**Table 3:** Key lessons from practice on perinatal management of neonates born to SARS-CoV-2 positive mothers.

1. COVID-19 in pregnancy was not associated with higher rates of preterm birth and intrauterine growth restriction.
2. There is no evidence to support intrauterine vertical transmission of SARS-CoV-2 from infected mothers as their offsprings were all negative and asymptomatic at birth.
3. Breast milk appears to be safe and protective for the neonate.
4. Skin-to-skin, bonding and breastfeeding may expose neonates to a higher risk of infection through contact or droplets. To reduce the risk of horizontal transmission, mother and care givers should adopt adequate preventive measures, such as frequent washing of hands, wearing surgical mask when handling or breastfeeding the baby.
5. Neonates who tested positive for COVID-19 within the first week, remained asymptomatic and in good health.
6. Neonates who tested positive for COVID-19 turned to negative within 1 week. On day 14 after birth they still tested negative.
7. As COVID-19 mothers have to undergo a very stressful quarantine period, we believe that bonding, rooming-in and breastfeeding would also help lessen this psychological burden.

## Data Availability Statement

All datasets presented in this study are included in the article/**Supplementary Material**.

## Ethics Statement

The study involving human participants was reviewed and approved by the local Ethics Committee (Comitato Etico dell'Area Vasta Emilia Nord). Newborns' parents provided their written informed consent to participate in this study. The Authors obtained written informed consent for publication from the newborns' parents.

## Author Contributions

GB and GC conceived the paper. BB, AR, CP, GV, RS, and RB provided and collected clinical and laboratory data and provided comments. GB, GC, AR, and MC wrote the first draft of the manuscript. All the authors have revised and approved the final version, were involved in the diagnosis, and perinatal management of mothers and/or neonates.

## Conflict of Interest

The authors declare that the research was conducted in the absence of any commercial or financial relationships that could be construed as a potential conflict of interest.
